# Bipolar disorder triggered by Covid-19 infection

**DOI:** 10.47626/2237-6089-2021-0430

**Published:** 2023-10-31

**Authors:** Marilene Costa, Marina Roman Meller, Flávio Kapczinski

**Affiliations:** 1 Universidade Federal do Maranhão São Luís MA Brazil Universidade Federal do Maranhão, Maranhão (UFMA), São Luís, MA, Brazil.; 2 Departamento de Psiquiatria Universidade Federal de São Paulo São Paulo SP Brazil Departamento de Psiquiatria, Universidade Federal de São Paulo (UNIFESP), São Paulo, SP, Brazil.; 3 Department of Psychiatry and Behavioural Neurosciences McMaster University Hamilton ON Canada Department of Psychiatry and Behavioural Neurosciences, McMaster University, Hamilton, ON, Canada.; 4 Instituto Nacional de Ciência e Tecnologia Translacional em Medicina Porto Alegre RS Brazil Instituto Nacional de Ciência e Tecnologia Translacional em Medicina (INCT-TM), Porto Alegre, RS, Brazil.; 5 Programa de Transtorno Bipolar Laboratório de Psiquiatria Hospital de Clínicas de Porto Alegre Porto Alegre RS Brazil Programa de Transtorno Bipolar, Laboratório de Psiquiatria, Hospital de Clínicas de Porto Alegre (HCPA), Porto Alegre, RS, Brazil.; 6 Departamento de Psiquiatria Universidade Federal do Rio Grande do Sul Porto Alegre RS Brazil Departamento de Psiquiatria, Universidade Federal do Rio Grande do Sul (UFRGS), Porto Alegre, RS, Brazil.

Neuropsychiatric manifestations have been widely reported after Coronavirus disease 2019 (COVID-19).^1,2^ Anxiety, depression, sleep disturbances, and post-traumatic stress disorder (PTSD) have been reported in 30-40% of COVID-19 survivors.^3,4^ Cases of reactive psychosis were also described.^5,6^ However, fewer cases of bipolar disorder (BD) after COVID-19 have been reported so far.

On 1st February 2021, a 47-year-old male afro-descendant, presented with flu-like symptoms, fever, and myalgia. Eleven days later, he was admitted to the hospital with fever and low oxygen saturation. The diagnosis of COVID-19 was confirmed by positive polymerase chain reaction (PCR) test. A computed tomography (CT) chest scan showed involvement of 50% of the lungs. He received oral azithromycin and oxygen therapy. One day later, his clinical condition deteriorated, he showed mental confusion, and a CT scan showed 80% pulmonary impairment, so he was transferred to an intensive care unit. Intravenous ceftriaxone and dexamethasone were added, along with supportive care. His clinical condition improved quickly and he was discharged after 5 days.

After hospital discharge, he presented with increased, loud, and accelerated speech, racing thoughts, psychomotor agitation, and an expansive and irritable mood. He complained angrily, arguing with people everywhere, including in traffic. He also showed belligerent behavior (e.g., he sued the hospital and his workplace) and low tolerance for noise and frustration. He spent so much money that he had to put his car and apartment up for sale. These behaviors represented a notorious change from his baseline personality, since he had always been a sensible person, restrained in his speech and actions.

The patient was diagnosed with bipolar disorder and quetiapine plus valproate were prescribed. He continued to act aggressively and overspend, so valproate was increased. A week later, the patient presented with depressive mood, frequent crying, anguish, low self-esteem, ruminations, feelings of guilt, excessive fear, decreased energy, social withdrawal, avolition, cognitive impairment (memory and concentration), and loss of interest in activities he enjoyed. Risperidone was added, but since the patient complained of sialorrhea, nightmares, and psychomotor agitation, it was replaced with aripiprazole. Since the depressive symptoms persisted, mirtazapine was added along with psychotherapy. The patient showed improvement in his psychiatric condition, maintaining mild depressive symptoms.

The patient’s previous medical history included a moderate anxiety disorder with depressive symptoms that had first been diagnosed 13 years previously. He had been treated with antidepressants, with full recovery from symptoms, and was followed up for more than a decade by the first author, never showing any manic symptoms. His family history was positive for anxiety disorder but negative for BD, schizophrenia, or psychosis. The patient is highly educated and works as a federal employee. He is married and has a son. Magnetic resonance imaging (MRI) of the brain showed no acute intracranial pathology or evidence of encephalitis. Laboratory investigations after discharge from the hospital were normal.

In this editorial, we present the case of a man who first developed manic and then depressive symptoms after a moderate SARS-CoV-2 infection. Unlike depressive and anxious symptoms that are commonly observed after COVID-19 infection,^4^ there are few reports of bipolar disorder symptoms. Four case reports describe onset of manic symptoms associated with COVID-19.^7-10^ All of these four patients were men, required hospitalization, and presented with grandiose elements in psychopathological assessment. None of them had exhibited depressive symptoms by the time the cases were published. Two of them had been given corticosteroids and antibiotics before the onset of manic symptoms, but time-effect and dose-response correlations made these medications less likely to be the trigger of mania in both cases.^7,9^ In two cases, cerebrospinal fluid (CSF) analysis was performed.^7,8^ In one of these, a mild pleocytosis was present and a SARS-CoV-2 specific IgG antibody test was positive, while reverse transcriptase PCR of CSF was negative.^7^ In the other case, all CSF parameters were normal.^8^ Regarding neuroimaging, one patient showed small ischemic lesions located at the basal ganglia and semiovale centrum on brain MRI,^7^ and two others showed no abnormalities.^8,9^

The short period of treatment, the duration of symptoms and the dose-response correlation made use of corticosteroids and antibiotics unlikely to be the trigger of manic-like symptoms. Since our patient had no personal or family history of BD and given that he presented with mental confusion before the onset of other symptoms, COVID-19 related brain damage is a possible etiology. However, a hypothesis of primary psychiatric disorder is relevant and cannot be excluded.

Neuropsychiatric manifestations are widely observed in individuals who have had COVID-19, with estimated incidence of 33.62% in the first 6 months after the acute infection.^3^ The risk of developing a neuropsychiatric manifestation is higher in, but not limited to, patients after a severe infection.^3^ However, neuropsychiatric symptoms can emerge even after mild cases, in the absence of respiratory insufficiency.^11^

At the beginning of the COVID-19 pandemic, it was still unclear whether this viral infection could damage the central nervous system (CNS).^7^ Currently, it is suggested that independent brain damage can occur along with SARS-CoV-2.^11^ It is also suggested that the brain involvement persists after respiratory symptoms are solved.^12^

There are various mechanisms that could explain how COVID-19 infection results in neuropsychiatric symptoms.^11^ By entering cells through angiotensin-converting enzyme 2 receptors, the virus can damage vascular endothelium in brain capillaries, resulting in thrombotic events and neuroinflammation.^13^ Increased levels of systemic proinflammatory cytokines (‘’cytokine storm’’), along with the altered coagulation cascade, lead to local microglia activation, which in turn may impair neurotransmission^14^ and cause neuronal damage.^11,15^ Although it appears to be uncommon, some authors also suggest direct viral invasion of the CNS^16-18^ can occur at the blood brain barrier^19^ or via circumventricular organs.^13^

This case highlights the importance of assessing presence of psychiatric symptoms in patients with COVID-19 infection. Early diagnosis and treatment can change patients’ outcomes and quality of life.
